# Association of the *KiSS1* gene with litter size in Cyprus and Iraqi black goats

**DOI:** 10.14202/vetworld.2021.1995-2001

**Published:** 2021-08-03

**Authors:** M. A. Rahawy, Hayder Abdul-Kareem AL-Mutar

**Affiliations:** 1 Department of Surgery and Theriogenology, College of Veterinary Medicine, University of Mosul, Mosul, Iraq; 2 Department of Surgery and Obstetrics, College of Veterinary Medicine, University of Baghdad, Iraq

**Keywords:** DNA sequence, goat, kisspeptin gene, litter size, polymorphism

## Abstract

**Aim:**

The study investigated the genetic polymorphism of the kisspeptin *(KiSS1)* gene and its relationship with litter size in Cyprus and Iraqi black goats.

**Materials and Methods:**

Blood samples (n=124) were collected from the two goat breeds reared at the Agricultural Research-Ruminant Research Station Breeding Station, Baghdad, Iraq. Genomic DNA was isolated using a DNA extraction kit. Polymerase chain reaction (PCR) was used to amplify the *KiSS1* gene. All PCR products were sequenced and samples were used for further analysis using NCBI-Blast online on the exon 1 (595 bp) region of the *KiSS1* gene.

**Results:**

The results of this study revealed a significantly (*P*<0.05) larger litter size of the Cyprus goat breed than in the Iraqi black goats in the first and second parity. Three (893G/C, 973C/A, and 979T/G) substitutions relative to the *KiSS1* gene reference sequence (GenBank ID: J × 047312.1, KC989928.1) were identified. Only the mutation g893G>C was identified as a single nucleotide polymorphism (SNP) associated with litter size. Furthermore, the average alleles in *KiSS*1 gene of both types of goats 0.567 and 0.3715 GG, were recorded. The genotyping at locus g893C>G was demonstrating domination of fecundity quality litter size, Both genotypes SNP of GC were classified at this marked region of *KiSS1* gene.

**Conclusion:**

The study concluded that the role of the *KiSS*1 gene in fecundity, revealing the status of this gene as an indicator in the assisted of caprine breeding selection.

## Introduction

Many genomic studies have been dedicated to identifying genes with economically significant polymorphisms. Researchers have shown that litter size can be determined genetically [1,2]. At present, in the field of genetics, there are ongoing efforts to identify candidate genes with dependable effects on continuous traits [3]. Litter size is a complicated factor that is economically important within caprine production. Many genetic markers have been shown to be associated with goat litter size. However, little has been revealed regarding the main genes related to litter size in caprine, but litter size appears to be controlled by multiple genes.

The previous studies [3,4] showed that the kisspeptin *(KiSS1)* gene makes a major contribution to multiparity in goats. The *KiSS1* gene is situated on the long arm of chromosome 1 (1q32) [5,6] and encodes the *KiSS1* (formerly known as metastin) protein [6]. G protein is connected with *KiSS1* gene receptor in the cells [5]. *KiSS1*/GPR54 signaling plays an essential role in the mechanism of initiation of hormone release by GnRH [7-9]. The *KiSS1* hormone is currently critical for monitoring fertility given its role in controlling physiological reproductive status [10-12]. Polymorphisms of the *KiSS1* gene have been shown to be associated with reproductive traits (such as high prolificacy, sexual precocity, and year-round estrus phenotypes of goats), suggesting its importance as a regulator of puberty onset [13]. Particularly in females, the negative and positive feedback loops of gonadotropins, which play a substantial role in the generation of the pre-ovulatory LH surge, appear to be operated by the hypothalamic *KiSS1*/GPR54 system [3]. Many studies have revealed that the *KiSS1* gene could be strongly related to reproductive traits in goats [14]. The genetic improvement of polygenic traits could be achieved by marker-assisted selection, which has higher accuracy in estimating an animal’s genetic value [15].

The study investigated the genetic polymorphism of the *KiSS1* gene and its relationship with litter size in Cyprus and Iraqi black goats.

## Materials and Methods

### Ethical approval

The study was approved by the Department of Surgery and Theriogenology, College of Veterinary Medicine, University of Mosul, Iraq. Under reference number (S.E.C 2017, Sem.).

### Study period and location

The study was conducted from March 2019 to March 2020 on Cyprus and Iraqi black goat breeds in the state slat for farming and Ruminant Researches Station - Ministry of Agriculture (Agurgof) Baghdad.

### Animals

This study was conducted on 124 animals, including 62 Cyprus goats and 62 Iraqi black goats. Goats from the two breeds were kept under the same management conditions. The age of first kidding in Cyprus goats was 14.48±1.34 months, with a kidding interval of 6.85±0.122 months. In contrast, the age of first kidding in Iraqi black goats was 16.37±0.127 months, with a kidding interval of 7.76±0.217 months. In order to determine the estrus and get successful copulation, the studied female goats were mixed with males of the same species and breed to ensure that no variations in the breed will interfere with the pregnancy outcomes. The reproductive records were conserved by veterinarians, and the litter size records were obtained from first and second parity (2018, 2019). Selection for litter size and also fertility traits had been performed on this flock in previous years.

### Sample collection

Blood samples were collected from the jugular vein in a tube containing 10 ml of anticoagulant (EDTA) under sterile conditions. These samples were transferred to the laboratory using an icebox and stored in a deep freezer at -20°C until DNA extraction.

### DNA extraction and template preparation

**Figure-1:**
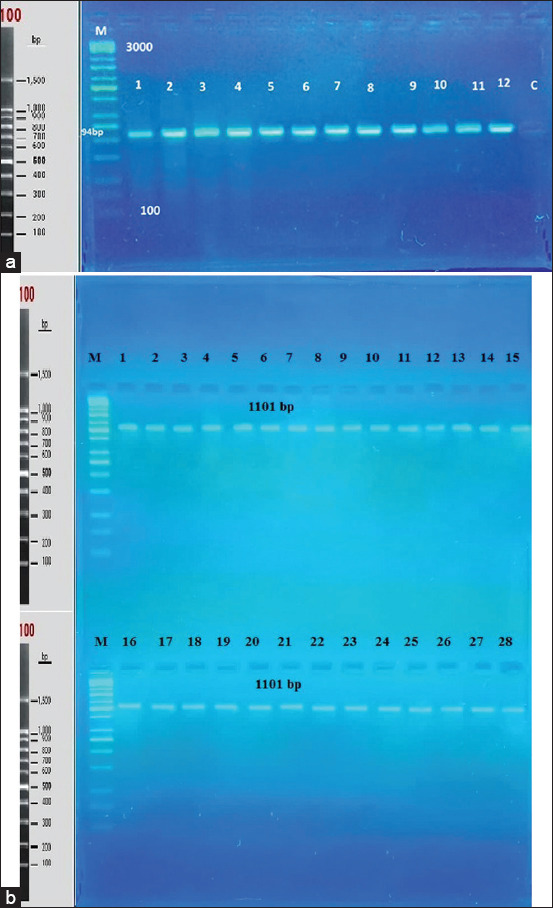
(a) Polymerase chain reaction (PCR) product of the organizer region of Kisspeptin-1 gene in the goat the band size 594 bp. with red stain The PCR produce was electrophoresis on 2% agarose (5 volt/cm^2^. 1×tris borate EDTA [TBE] buffer) for 1:30 h. C: Control, M: DNA ladder (100). (b): PCR product of the organizer region of Kisspeptin-1 gene in the goat the band size 1101 bp. with red stain The PCR product was electrophoresis on 2% agarose (5 volt/cm^2^. 1×TBE buffer) for 1:30 h. C: Control, M: DNA ladder (100).

In accordance with the manufacturer’s instructions, the DNA of all goats used in this study was isolated using a DNA extraction kit (G-Spin DNA Extraction, Intron Biotechnology, cat. no. 17045). The amplified product of the *KiSS1* gene was subjected to electrophoresis on 2% agarose gel, and a single clear band of 594 bp was obtained, which was compared with a marker of 3000 bp for confirmation (Figure-1).

### Polymerase chain reaction (PCR) reaction

*KiSS1* was identified by PCR amplification of the *KiSS1* gene. The reaction was carried out using a PCR reaction tube (Biozyme, Oldenhorf, Germany) with a total mixture of 10 μL. Two primer sequnances of *KiSS*1 gene as follow;

The first reaction consists of 1 μL of forward primer (5′-TCTTCTCTCCTGGGATCGGG-3′) and reverse primer (5′-GCCCAGAGAGAGGCTTTGG-3′) (1101 bp) and second reaction was consist of forward primer (5-TGCAAAGCCGAGTGTGCAGG-3) and reverse primer (5TGAAGGCGGTGGCACAAAGG-3) (594 bp), (each 10 pmol/μL), according to An *et al*. [3]. The *KiSS1* gene (each 10 pmol/ml) (IDT/Canada), with a molecular weight of 594 bp, 5 μL of 2×Go Taq PCR Pre-Mix, and 16.5 μL of nuclease-free water. Finally, 1.5 μL of DNA template was added to each reaction tube (Intron, Canada; Cat. No. 25025). Amplification conditions were as follows: Denaturing at 95°C for 3 min; 35 cycles of denaturation (95°C for 4 s), annealing (60°C for 4 s), and extension (72°C for 45 s); and final extension at 72°C for 7 min. The amplicons were determined by gel electrophoresis together with a DNA marker 100 bp ladder (Ladder Marker 100 bp, cat. no. KK6302; Kapa, USA) in 2% agarose gel (cat. no. 8100.11; Conda, USA). PCR products were visualized using electrophoresis on 2% (w/v) agarose in parallel with 100 bp DNA markers. The PCR products were sequenced in both directions.

### DNA sequences

All samples (PCR products) from the primers (594 bp) obtained from the Cyprus and Iraqi black goat populations were used. The PCR product was sequenced using *Capra hircus* sequences (Sequence ID: J × 047312.1, KC989928.1), with online BLAST-NCBI and BioEdit.

### PCR-restriction fragment length polymorphism (RFLP)

The PCR-RFLP method was performed on the *KiSS1* gene. Amplification of 10 μL of PCR products was performed for primer 1101 bp with the AclI restriction enzyme (Thermo Scientific) (7). The incorporation reaction was conducted with 10 μL as final volume at 37°C for 4 h, for the digestion of PCR products.

### Statistical analysis

Statistical analysis of the data was performed using the statistical SPSS v.23 software (SPSS Inc. Chicago, IL., USA). The data were run using the Hardy–Weinberg equilibrium (HWE) test and Fisher’s exact test [16].

## Results

### Determination of litter size

Cyprus goats were 14.48±1.34 months old at first kidding, with a kidding interval of 6.85±0.122 months. Overall, 98 and 100 kids were born with litter sizes of 1.58±0.012 and 1.62±0.008 at first and second parity, respectively, Iraqi black goats were 16.37±0.127 months of age at 1^st^ kidding, with a kidding interval of 7.76±0.217 months. Overall, 79 and 81 kids were born with litter sizes of 1.29±0.017 and 1.31±0.021 at first and second parity, respectively (Table-1).

**Table 1:** SNPs and Amino acid change observed at polymorphic sites of Cyprus and local black goat.

*Capra hircus* breed Cyprus and Local black kisspeptin (KiSS-1) gene				

No. of sample	Nucleotide	Nucleotide change	Amino acid change	Predicted effect
Cyprus goat	G\T	No translate protein	UTR	No translate protein
	G\A	No translate protein	UTR	No translate protein
	T\C	No translate protein	UTR	No translate protein
	A\T	No translate protein	UTR	No translate protein
	G\A	No translate protein	UTR	No translate protein
	G\C	No translate protein	UTR	No translate protein
	C\T	No translate protein	UTR	No translate protein
	T\C	TGG\CGG	W\R	Missense
Local black goat	G\T	No translate protein	UTR	No translate protein
	G\A	No translate protein	UTR	No translate protein
	G\C	No translate protein	UTR	No translate protein
	C\A	No translate protein	UTR	No translate protein
	T\C	No translate protein	UTR	No translate protein
	C\T	AAC\AAT	N\N	silent
	G\A	GTG\ATG	V\M	Missense
	C\A	CTT\ATT	L\I	Missense
	T\G	TGG\GGG	W\G	Missense
	G\A	CAG\CAA	Q\Q	silent
	T\G	CTT\CTG	L\L	silent

### Sequencing of the *KiSS1* gene and association with litter size

**Figure-2:**
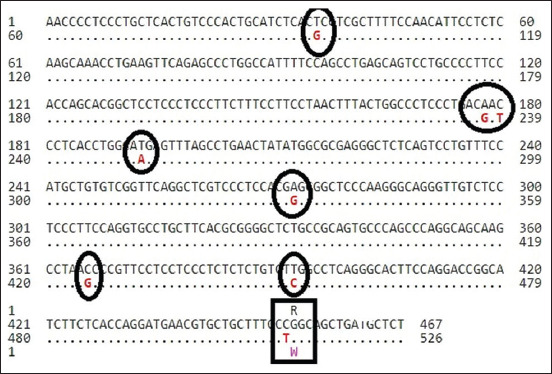
Alignment of nucleotide seq. *Capra hircus* kisspeptin (*KiSS1*) gene with gene bank of NCBI; appear agreement of gene in Sequence ID: K JX047312.1in Cyprus Goat.

**Figure-3:**
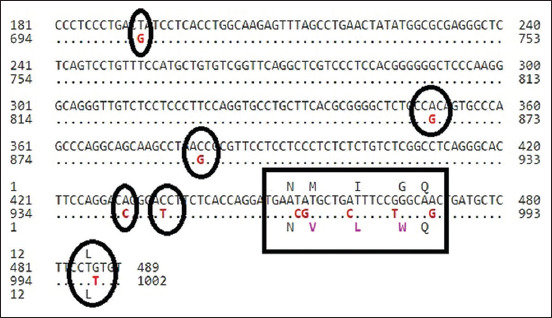
Alignment of nucleotide seq. *Capra hircus* kisspeptin (*KiSS1*) gene with gene bank of NCBI; appear agreement of gene in Sequence ID: KC989928.1in local black goat.

In this study, the sequence of primers used was linked to the exon 1 fragment of *KiSS1* gene in both studied breeds., Here, two goat breeds (n=124) were utilized to examine single nucleotide polymorphisms (SNPs) in the coding regions of exon 1 and the proximal of studied gene at 594 bp were run for DNA sequencing. The sequencing results revealed 98% compatibility with the normal *C. hircus* *KiSS1* gene. Three polymorphic sites (with transversion mutations) in exon 1 (893G>C, 973C>A, and 979T>G) were used as the reference sequence of gene (ID: JX047312.1 for Cyprus goat and KC989928.1 for Iraqi black goat samples) (Figures-2 and 3).

**Figure-4:**
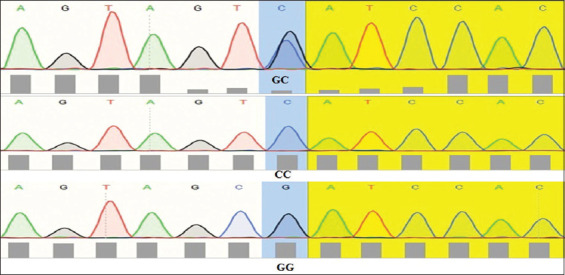
Wild-type and new variant G\C, C\C and G\G of exon I *KiSS1* gene.

**Figure-5:**
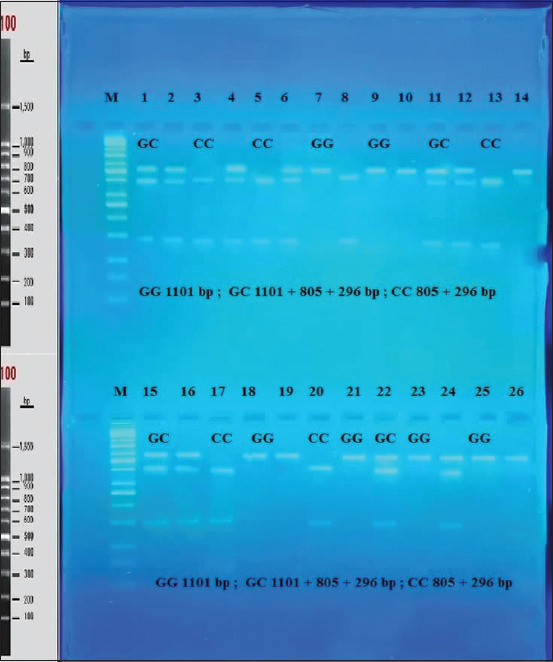
RE digestion (*Acl I*) of *KiSS1* gene of intron 1 and partial Intron 1 (1101bp) in agarose gel electrophoresis (2%).

Similarly, the remaining mutations at 973C>A and 979T>G did not contribute to the fitness and had no direct effect on litter size, although there were associations with changes in the amino acids (Table-1). As such, only mutation at locus g893G>C was measured for an association with litter size in the present study (Table-2). The SNP was shown to be associated with a difference in litter size in the Cyprus goat breed (Table-2). Cyprus goats revealed a significant difference (*P*<0.05) in litter size compared to the Iraqi black goats at the 1^st^ and 2^nd^ parity. A higher litter size (least-square means [LSM]±standard errors [SE]=1.62±0.008) was obtained in the 2^nd^ parity, while a lower one (LSM±SE=1.58±0.012) was in the 1^st^ parity. Similarly, in Iraqi black goats, a higher litter size (LSM±SE=1.31±0.021) was found in the 2^nd^ parity relative to that in the 1^st^ parity (LSM±SE=1.29±0.017). At the g.893G>C locus in Cyprus goats (Table-2), the genotype with nucleotides GG was higher in 1^st^ and 2^nd^ parity than CC nucleotides genotype. Meanwhile, in Iraqi black goats, relationship with GG genotype was higher in litter size than CC genotype in the 1^st^ and 2^nd^ parity average parity in Cyprus goat, associated with *GG* genotype greater litter size than an Iraqi black goat with *GG* genotype in the 1^st^ and 2^nd^ parity significantly at *P*<0.05. (Figure-4). After digestion of the amplified PCR fragments, GG and GC genotypes exhibited homozygotes and GC exhibited heterozygotes in both breeds of goats. (Table-2 and Figure-5).

**Table 2:** The litter size of Cyprus and local black goat at locus *g*.389 *G* >*C* square means and standard errors.

Locus	Goat breed	Genotype	Number of goat/Frequencies	Litter size with 1^st^ parity	Litter size with 2^ed^ parity	Average litter size
G/C	Cyprus [62]	GG	13/0.209	1.58±0.012a	1.62±0.008a	1.60±0.011a
		GC	32/0.516	1.32±0.009b	1.48±0.011b	1.40±0.010b
		CC	17/0.275	1.44±0.007b	1.48±0.013b	1.46±0.011b
	Black [62]	GG	8/0.130	1.29±0.017a	1.31±0.021a	1.30±0.020a
		GC	30/0.483	1.18±0.023b	1.26±0.014b	1.22±0.018b
		CC	24/0.387	1.23±0.024b	1.21±0.019b	1.22±0.021b

Different values within the same column differ significantly at p<0.05

### Submission of local Iraqi isolate to NCBI

The *C. hircus* *KiSS1* gene was registered with the National Center for Biotechnology Information with accession number: MT897468.1; MT897469.1; and MT897470.1. Ongoing work will add to this as data from more strain types are published and made available for download.

## Discussion

*KiSS1* is a product of the *KiSS1* gene, which plays an important role in reproductive functions, acting primarily on the gonadotropic axis at the level of the hypothalamus [3,17]. *KiSS1* is recognized as a natural ligand of an orphan G protein-coupled R (GPR54), and it is evident that the hypothalamic *KiSS1*/GPR54 system plays a central role in controlling the beginning of puberty by regulating the secretion of GnRH from hypothalamic neurons [5]. The results of this study showed that polymorphisms of the *KiSS1* gene cause an increase of the litter size in two goat breeds. The 1^st^ parity effect on genes was significantly higher (*P*<0.05) than 2^nd^ parity in Cyprus and Iraqi black goats. Our results are in agreement with a previous study in which the litter size of Cyprus goat breeds was recorded as 1.60 [18]. Meanwhile, the results of the present study showed that the litter size of Iraqi black goats in second parity was 1.30±0.020, which is in agreement with a previous study of Juma *et al*. [19], in which a litter size of 1.27±0.14 was recorded in black goats for the treatment with hormones groups in comparison with 1.16±0.16 in a control group in northern Iraq.

The genetic diversity of polymorphism content in combination with genotypes of different *KiSS1* gene loci was associated with litter size performed in both Cyprus and Iraqi black Doe at locus g. 893G>C. Significant (*P*<0.05) genetic diversity was determined at that locus in the HWE test in Cyprus and Iraqi black goats. It is suggested that mutation of this gene is related to high prolificacy in small ruminants [20].

The current study showed that there are three polymorphisms of the *KiSS1* gene in Cyprus and Iraqi black goats (893G>C, 973C>A, and 979T>G). The findings showed that the coding sequence of the *KiSS1* gene showed 98% similarity with *C. hircus*. The changes of amino acids were lysine to asparagine at g.893 G>C, cysteine to tryptophan at g.973C>A, and leucine to isoleucine at g.979 C>A. It appeared that the polymorphism in the *KiSS1* gene at locus G893C was significantly (*P*<0.05) associated with litter size in Iraqi black and Cyprus goat breeds. The present results regarding the *KiSS1* gene agree with a previous study in two Ethiopian goat breeds [21]. The mutation at g.895G>C is considered to be associated with litter size. The regulatory roles of the *KiSS1* gene may be similar between cattle and goats. All of the 893G>C, 973C>A, and 979T>G loci were linked in the gene of the two studied breeds, which may have resulted from the selection. The two SNP loci were not in HWE in Cyprus and local black breeds (*P*<0.05), indicating that the genotypic characteristics had been influenced by selection, mutation, or migration. Accumulating evidence further revealed that the central or peripheral administration of *KiSS1* stimulates GnRH-dependent LH and FSH production in all mammalian species [22]. *KiSS1* is the essential factor in governing reproductive functions in small ruminants. The biochemical and physiological functions, together with our results obtained in this study, show that the *KiSS1* gene could be used as a molecular breeding marker in goats [3,21].

In a previous study An *et al*. [3], it was shown that four SNPs (384G>A, 2489T>C, 2510G>A, and 2540C>T) potentially linked with litter size had been detected in Xinong Saanen goats and Guan Zhong goats, while no polymorphism was detected in exon 1 of the goat *KiSS1* gene [23]. In Egyptian small ruminant breeds, the loci g2124T>A and g2270C>T were found to be significantly related to litter size [4]. However, in Indian goats, the loci G296C, G2510A, and C2540T were associated with litter size [22]. In addition, in Black Bengal goat, the locus of the *KiSS1* gene and age at the start of puberty was linked with litter size [13]. In the same way, of the remaining polymorphisms, only g.893G>C considered in this study was found to be highly associated with litter size linked to GG than CC nucleotide genotypes in the goats. The highest percentage of GG nucleotide was noticed in exon1 regions (56.7% of Cyprus doe and 37.15% for Iraqi black doe, respectively). Therefore, the natural variety in short genes, such as *KiSS*1 gene [24]. Suggesting that may be generated by the increased *KiSS*1 output and sensitivity. All of these results indicate that the *KiSS1* gene is an excellent candidate gene for determining reproductive traits in animals [3]. Our study demonstrated that the genotypes made remarkable contributions (*P*<0.05) to litter size as a reflection of fecundity performance. The current study disagrees with the previous study [24] for the CC genotype of goat breed does the litter size was difference assessed for value 0.80 at 296 loci. Therefore, the *KiSS1* gene is considered to be an important candidate gene for reproductive traits in livestock and humans [3,25]. It has been suggested that the *KiSS1* gene plays a crucial role in reproductive performance in animal species [6,26,27]. It was previously reported [20] that six *KiSS1* gene polymorphisms were found in five goat breeds with different prolificacy. Moreover, recently, 11 SNPs were detected in three different goat breeds [3,28-31].

## Conclusion

Polymorphisms of the *KiSS1* gene play an important role in reproductive functions and have potential roles as genetic markers in caprine genotyping associated with reproductive traits. In addition, our results revealed the extent of the genetic variation of the *KiSS1* gene in caprine, which could help to improve the genetic breeding of goats.

## Authors’ Contributions

MAR and HAA: Contributed in the conceptualization. HAA: Collected the samples, conducted the laboratory examinations, DNA Extraction, PCR Reaction, DNA Sequences, and PCR-RFLP. MAR: Performed data organizationand data analysis and drafted and edited the manuscript. All authors read and approved the final manuscript.
